# First-line biologic and targeted synthetic DMARDs versus methotrexate in early rheumatoid arthritis: a meta-analysis of remission outcomes

**DOI:** 10.1093/rheumatology/keag515

**Published:** 2026-09-17

**Authors:** Mark D Russell, Chun J Chan, Aran Sasitharan, Victoria Allen, Mark Gibson, Maryam A Adas, Samantha Goodliffe, Katie Bechman, Andrew P Cope, Sam Norton, Ailsa Bosworth, Peter Foxton, Elizabeth Price, Sana Sharrack, Shouvik Dass, Paul Emery, Lesley Kay, Peter Lanyon, James B Galloway

**Affiliations:** Centre for Rheumatic Diseases, King’s College London, London, UK; Centre for Rheumatic Diseases, King’s College London, London, UK; Centre for Rheumatic Diseases, King’s College London, London, UK; Centre for Rheumatic Diseases, King’s College London, London, UK; Centre for Rheumatic Diseases, King’s College London, London, UK; Centre for Rheumatic Diseases, King’s College London, London, UK; Centre for Rheumatic Diseases, King’s College London, London, UK; Centre for Rheumatic Diseases, King’s College London, London, UK; Centre for Rheumatic Diseases, King’s College London, London, UK; Centre for Rheumatic Diseases, King’s College London, London, UK; National Rheumatoid Arthritis Society, Maidenhead, UK; National Rheumatoid Arthritis Society, Maidenhead, UK; Rheumatology Department, Great Western Hospital NHS Foundation Trust, Swindon, UK; Leeds Institute of Rheumatic and Musculoskeletal Medicine, University of Leeds, Leeds, UK; NIHR Leeds Biomedical Research Centre, Leeds Teaching Hospitals NHS Trust, Leeds, UK; NHS England, London, UK; Rheumatology Department, Leeds Teaching Hospitals NHS Trust, Leeds, UK; Leeds Institute of Rheumatic and Musculoskeletal Medicine, University of Leeds, Leeds, UK; NIHR Leeds Biomedical Research Centre, Leeds Teaching Hospitals NHS Trust, Leeds, UK; NHS England, London, UK; Rheumatology Department, Newcastle upon Tyne Hospitals NHS Foundation Trust, Newcastle, UK; NHS England, London, UK; Rheumatology Department, Nottingham University Hospitals NHS Trust, Nottingham, UK; Centre for Rheumatic Diseases, King’s College London, London, UK

**Keywords:** biologic, JAK inhibitor, methotrexate, remission, meta-analysis, systematic review

## Abstract

**Objectives:**

To evaluate initial therapy with biologic and targeted synthetic DMARDs (b/tsDMARDs), compared with methotrexate, in patients with early RA.

**Methods:**

We systematically reviewed and meta-analysed randomized-controlled trials comparing initial b/tsDMARDs, combined with methotrexate or as monotherapy, against methotrexate in methotrexate-naïve adults with early RA. Pairwise and network meta-analysis were performed to compare DAS28 remission attainment, with the primary analysis at 6 months and secondary analyses at 3 and 12 months. Secondary outcomes included ACR50 response and CDAI remission.

**Results:**

Twenty-nine trials (13 387 participants) were included. Compared with methotrexate alone, initial b/tsDMARDs plus methotrexate significantly increased DAS28 remission at 6 months (risk ratio [RR] 1.62; 95% CI: 1.39, 1.89), 3 months (RR 1.95; 1.47, 2.57) and 12 months (RR 1.54; 1.39, 1.71), with benefits also observed for CDAI remission and ACR50 response. In network meta-analysis, all b/tsDMARD classes combined with methotrexate had higher remission rates than methotrexate alone. The benefits of b/tsDMARDs plus methotrexate were greater in studies with higher baseline disease activity and a greater proportion of rheumatoid factor-positive participants. As monotherapy, b/tsDMARDs increased DAS28 remission attainment compared with methotrexate at 6 months (RR 1.81; 1.42, 2.31), 3 months (RR 1.92; 1.31, 2.83) and 12 months (RR 1.46; 1.25, 1.71); however, at individual class level, significantly higher remission rates were observed only with tocilizumab and Janus kinase (JAK) inhibitor monotherapy.

**Conclusion:**

Initial therapy with b/tsDMARDs in combination with methotrexate is associated with higher remission rates than methotrexate alone in patients with early RA. As monotherapy, higher remission rates were observed for JAK inhibitors and tocilizumab.

*Rheumatology* key messages

- Initial therapy with b/tsDMARDs plus methotrexate is associated with significantly higher remission rates than methotrexate alone in early RA.
- As monotherapy, higher remission rates were observed for JAK inhibitors and tocilizumab compared with methotrexate.
- In meta-regression analyses of study-level data, higher baseline DAS28 and seropositivity were associated with greatest benefit from early b/tsDMARDs combined with methotrexate.

## Introduction

Biologic and targeted synthetic DMARDs (b/tsDMARDs) have transformed outcomes for rheumatoid arthritis (RA) and many other immune-mediated inflammatory diseases. Large reductions in acquisition costs in the era of biosimilars have created the potential for earlier use of b/tsDMARDs in treatment pathways. Recently updated EULAR guidelines recommend escalation to b/tsDMARDs for individuals with RA who have failed their initial conventional synthetic DMARD (csDMARD) therapy [1]. In the UK, NICE guidance permits escalation to b/tsDMARDs for people with moderate or severe RA who have responded inadequately, or been intolerant to, at least two csDMARDs [2].

Despite advances in therapies, substantial barriers remain to earlier use of b/tsDMARDs in routine clinical practice. The requirement for treat-to-target monitoring and escalation of csDMARDs can mean that patients who would benefit from earlier b/tsDMARD initiation are often not escalated for several months or years after diagnosis [3, 4]. In England and Wales, only 9% of patients newly diagnosed with RA in 2022 were escalated to b/tsDMARDs within a year of diagnosis [3], and rates of remission for RA have been static since 2018 [5]. Remission attainment in the UK and several other European cohorts remains substantially below the levels achieved in trials of early b/tsDMARD initiation (with >70% Disease Activity Score in 28 joints [DAS28] remission achieved in some studies) [6, 7]. Remission rates are even lower for patients with RA who have well-recognized adverse prognostic factors, including high disease activity at diagnosis and seropositivity [5, 8–11].

To improve outcomes for patients with RA, particularly those with poor prognostic factors at diagnosis, it has been suggested that b/tsDMARDs should form part of the initial treatment strategy [12]. Our objective was to perform a systematic review and meta-analysis to evaluate the efficacy of initial b/tsDMARD therapy, compared with methotrexate, in patients with early RA who have not previously been treated with methotrexate.

## Methods

### Search strategy and eligibility criteria

A systematic literature search was undertaken in the MEDLINE, Embase and Cochrane CENTRAL databases to identify randomized controlled trials (RCTs) of b/tsDMARDs *vs* methotrexate in participants with early RA who had not previously been treated with methotrexate. The search was performed from database inception to 23 June 2026 using disease, drug and study-related terms as specified in the Supplementary Material. Previously published reviews and reference lists were screened to identify additional eligible studies. The search was performed in accordance with the Preferred Reporting Items for Systematic Reviews and Meta-Analyses guidance [13], and registered with the International Prospective Register of Systematic Reviews (PROSPERO registration ID: CRD420261436552).

Studies were eligible for inclusion if they were RCTs evaluating the initiation of b/tsDMARDs, either in combination with methotrexate or as monotherapy, against a methotrexate-based comparator in adults with early RA, defined as a mean disease duration of 3 years or less. To be eligible for inclusion, participants had to be methotrexate-naïve or have received methotrexate for <4 weeks before enrolment. Studies including participants with early RA previously treated with csDMARDs other than methotrexate (e.g. hydroxychloroquine or sulfasalazine) were eligible for inclusion. In addition, studies were required to report remission outcomes using DAS28 at one or more pre-defined time-points (3, 6 or 12 months post-randomization). Where these outcomes were not reported in the primary publication, other publications and conference abstracts from the same study were searched, in addition to clinicaltrials.gov, to identify the relevant data where available. In studies evaluating multiple doses of the same b/tsDMARD, only doses licensed for the treatment of RA were included.

### Study selection, data extraction and bias assessment

Records identified through the search strategy were screened independently by two reviewers (C.J.C. and A.S.). Titles and abstracts were assessed initially, followed by full-text review of potentially eligible articles. Any disagreements regarding eligibility were resolved through consultation with a third reviewer (M.D.R.). Data extraction was performed independently by two reviewers (C.J.C. and M.D.R.) using a standardized data collection form. Extracted variables included study characteristics, participant demographics, baseline disease activity, serological status, intervention and comparator regimens, and efficacy outcomes at 3, 6 and 12 months (defined below). Risk of bias was assessed independently by two reviewers (C.J.C. and M.A.A.) using the Cochrane Risk of Bias-2 tool. Assessments considered the five RoB 2 domains, with each domain and the overall result judged as low risk, some concerns, or high risk of bias (see Supplementary Material); bias due to missing outcome data and selection of the reported result was assessed according to the RoB 2 signalling questions and guidance [14]. Discrepancies were resolved by consensus, with involvement of a third reviewer where necessary (M.D.R.).

### Outcomes

The primary outcome of interest was DAS28 remission, defined as a DAS28 < 2.6. DAS28 remission was chosen *a priori* as the primary outcome as it is commonly used in clinical practice to assess disease activity and treatment response in individuals with RA, and is frequently reported as an efficacy outcome in clinical trials. Six months post-randomization was selected as the primary end point, because treatment strategies frequently diverged beyond this point due to rescue therapy, treatment escalation and withdrawal protocols. DAS28 remission attainment at 3 and 12 months post-randomization were reported as secondary outcomes. DAS28-CRP was prioritized when both DAS28-CRP and DAS28-ESR remission data were reported. DAS28-ESR data were used when CRP-based outcomes were not reported. Additional secondary outcomes included the American College of Rheumatology 50% response criteria (ACR50) and Clinical Disease Activity Index (CDAI) remission (defined as a CDAI score ≤2.8), where available.

### Statistical analysis

Pairwise meta-analysis was conducted to estimate pooled risk ratios (RRs) and 95% CIs for DAS28 remission attainment at 6 months, and separately at the 3- and 12-month secondary timepoints. Secondary analyses included ACR50 response at 6 months and CDAI remission attainment at 6 months.

Separate pairwise meta-analyses were performed to compare: (i) b/tsDMARDs in combination with methotrexate *vs* methotrexate-based comparators; and (ii) b/tsDMARD monotherapy *vs* methotrexate-based comparators. Treatment regimens were permitted to include additional csDMARDs and/or corticosteroids alongside methotrexate. Pooled effect-estimates were calculated using DerSimonian and Laird random-effects models. No continuity corrections were required because no studies contained zero-event cells. Statistical heterogeneity was quantified using the *I*² statistic and between-study variance (τ²). *I*² was interpreted as low (0–40%), moderate (30–60%), substantial (50–90%) or considerable (75–100%) [15].

To investigate potential sources of treatment effect heterogeneity between studies, random-effects meta-regression was performed using aggregated, study-level covariates, including mean participant age, sex distribution, rheumatoid factor positivity, anti-cyclic citrullinated peptide (CCP) positivity, mean DAS28 score at enrolment, baseline corticosteroid use and year of study publication. Publication bias and small-study effects were explored through visual inspection of funnel plots and Egger’s regression test.

Several sensitivity analyses were conducted to assess the consistency of the primary pairwise meta-analysis findings, including: (i) exclusion of studies in which comparator groups were randomized to receive methotrexate in combination with other csDMARDs and/or corticosteroids; (ii) leave-one-out analyses, which evaluate the influence of individual studies on pooled estimates; (iii) restriction to studies enrolling participants without prior exposure to csDMARD therapy; and (iv) excluding studies deemed at high risk of bias in at least one domain.

Additionally, network meta-analysis was performed to explore outcome attainment between b/tsDMARDs from different classes (TNF inhibitors [TNFi], Janus kinase inhibitors [JAKi], IL-6 inhibitors, CD20 inhibitors and T cell co-stimulation modulators), incorporating both direct and indirect evidence from available studies. Separate network meta-analyses were performed to compare: (i) b/tsDMARDs in combination with methotrexate *vs* methotrexate-based comparators; and (ii) b/tsDMARD monotherapy *vs* methotrexate-based comparators. Random-effects models were fitted using the *network* and *mvmeta* packages in Stata, with restricted maximum likelihood estimation assuming a common between-study heterogeneity variance across treatment comparisons. Treatment effects were reported as RRs with 95% CIs. For trials with multiple eligible intervention arms, each comparison was included separately. The structure of the evidence network was illustrated using network plots. Due to the limited numbers of direct head-to-head comparisons between b/tsDMARD classes, results were presented only for comparisons with methotrexate-based therapy; comparisons between individual b/tsDMARD classes and treatment rankings were not presented for this reason. Consistency between direct and indirect evidence was assessed using a global Wald test for inconsistency and node-splitting analyses for individual treatment comparisons [16, 17]. To explore whether differences between b/tsDMARD classes contributed to between-study heterogeneity, additional network meta-analyses were performed in which all b/tsDMARDs within each comparison were combined into a single treatment node, and heterogeneity estimates (τ) were compared with those from class-specific models.

All analyses were performed using Stata version 19 (StataCorp, College Station, TX, USA).

### Ethical approval

This study used only publicly available data. As per UK Health Research Authority guidance, no ethical approval was therefore required.

### Patient and public involvement

Individuals with lived experience of RA were involved in interpreting the study findings, reviewing and co-authoring the manuscript. The findings of this study will be disseminated with the involvement of patients and leading patient organizations for individuals with RA.

## Results

### Study characteristics

From 4780 records identified from database searches, 29 studies (13 387 participants) were included, comparing b/tsDMARDs with methotrexate in methotrexate-naïve patients with early RA (Supplementary Fig. S1). Twenty-six studies contributed to the comparison of b/tsDMARDs plus methotrexate *vs* methotrexate alone, with 28 b/tsDMARD arms in total (17 TNFi, three JAKi, three tocilizumab, four abatacept and one rituximab arm). Eleven studies contributed to the comparison of b/tsDMARD monotherapy *vs* methotrexate, with 11 b/tsDMARD arms in total (five JAKi, three tocilizumab, two TNFi and one abatacept arm). Details of included studies are shown in Supplementary Table S1.

Across eligible studies, the participant-weighted mean age at enrolment was 50.8 years (between-study standard deviation [s.d.] 2.1); 75.7% of participants were female (s.d. 5.0); 85.7% were rheumatoid factor positive (s.d. 10.4); 81.8% were CCP positive (s.d. 9.4); the mean baseline DAS28 was 6.03 (s.d. 0.61); and 38.1% were receiving corticosteroids at enrolment (s.d. 14.1). Six of 29 (20.7%) eligible studies were deemed at low risk of bias across all domains; 19 (65.5%) studies were considered to have some potential sources of bias; and 4 (13.8%) studies had at least one domain with high risk of bias (Supplementary Table S2).

Data on DAS28 remission attainment at 6 months post-randomization were available from 17 b/tsDMARD plus methotrexate study arms, 10 b/tsDMARD monotherapy arms, and 20 methotrexate arms: crude participant-weighted remission attainment at 6 months was 47.9%, 40.5% and 25.9%, respectively. Corresponding remission data at 3 months were available from 11 b/tsDMARD plus methotrexate arms, five b/tsDMARD monotherapy arms and 12 methotrexate arms, with remission attainment in 39.9%, 34.3% and 18.9%, respectively. At 12 months, data were available from 23 b/tsDMARD plus methotrexate arms, nine b/tsDMARD monotherapy arms and 25 methotrexate arms, with remission attainment in 47.0%, 37.7% and 28.7%, respectively.

### Pairwise meta-analysis

For the primary outcome at 6 months post-randomization, participants assigned to b/tsDMARDs plus methotrexate were 62% more likely to achieve DAS28 remission than those assigned to methotrexate-based therapy alone (risk ratio [RR] 1.62; 95% CI: 1.39, 1.89; *n* = 17 studies; Fig. 1A). DAS28 remission attainment was also higher at 3 months (RR 1.95; 95% CI: 1.47, 2.57; *n* = 11 studies; Supplementary Fig. S2) and 12 months post-randomization (RR 1.54; 95% CI: 1.39, 1.71; *n* = 23 studies; Supplementary Fig. S3). In secondary analyses, ACR50 response at 6 months was higher for participants assigned to b/tsDMARDs plus methotrexate than for methotrexate alone (RR 1.37; 95% CI: 1.23, 1.52; *n* = 14 studies; Supplementary Fig. S4). Significant benefit was also observed using CDAI remission criteria (RR 1.75; 95% CI: 1.25, 2.44; *n* = 5 studies; Supplementary Fig. S5), albeit with fewer studies reporting this outcome.

**Figure 1:**
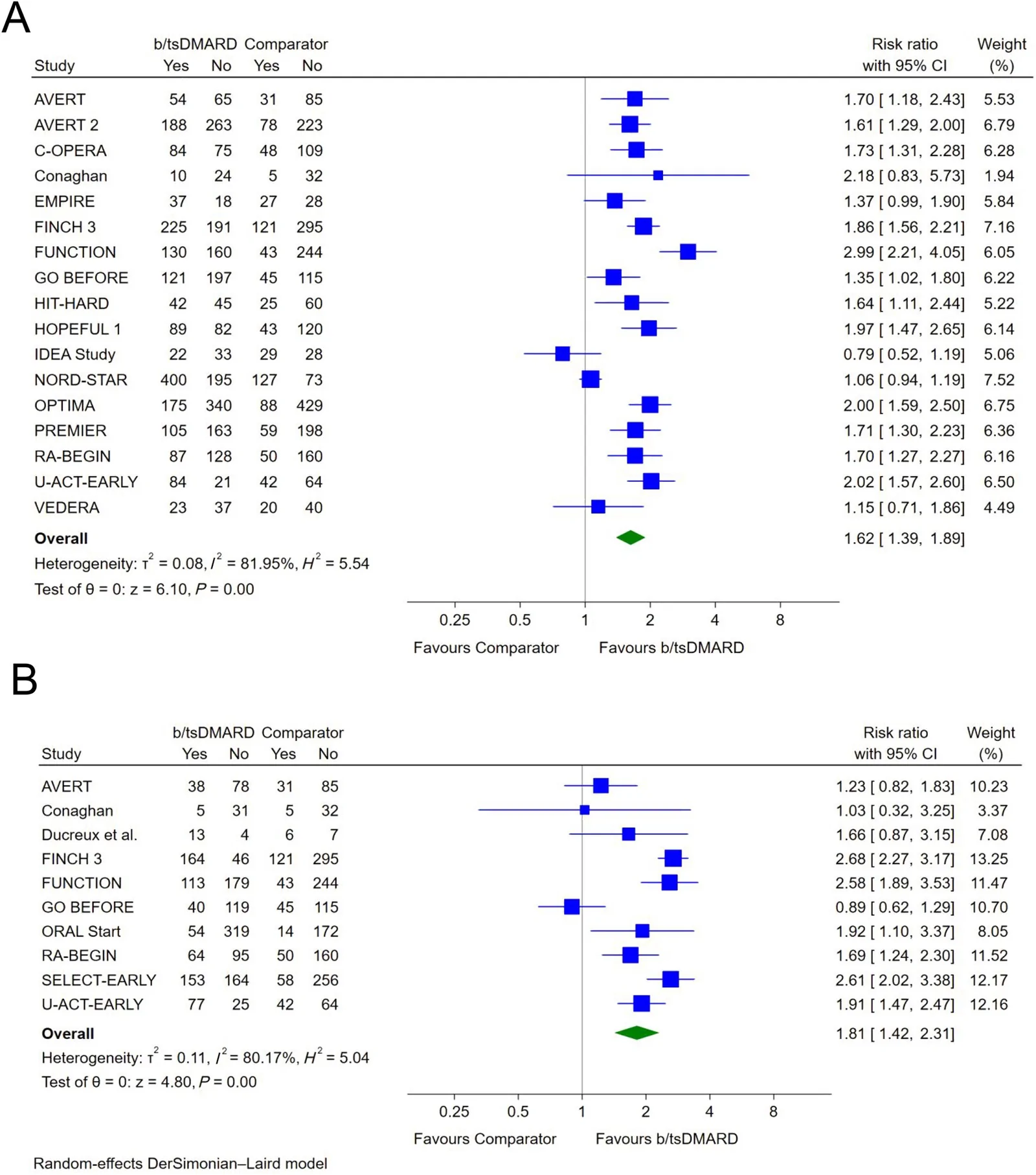
Pairwise meta-analysis of DAS28 remission attainment at 6 months post-randomization in methotrexate-naïve participants with early RA, comparing b/tsDMARDs plus methotrexate *vs* methotrexate-based therapy (**A**, top panel), and b/tsDMARD monotherapy *vs* methotrexate-based therapy (**B**, bottom panel). b/tsDMARDs: biologic and targeted synthetic DMARDs; DAS28: Disease Activity Score in 28 joints.

Similar findings were observed for b/tsDMARD monotherapy. At 6 months, participants assigned to b/tsDMARD monotherapy were 81% more likely to achieve DAS28 remission than those assigned to methotrexate (RR 1.81; 95% CI: 1.42, 2.31; *n* = 10 studies; Fig. 1B). DAS28 remission attainment was also higher at 3 months (RR 1.92; 95% CI: 1.31, 2.83; *n* = 5 studies; Supplementary Fig. S6) and 12 months (RR 1.46; 95% CI: 1.25, 1.71; *n* = 9 studies; Supplementary Fig. S7). In secondary analyses, participants assigned to b/tsDMARD monotherapy had higher ACR50 responses at 6 months (RR 1.51; 95% CI: 1.30, 1.75; *n* = 7 studies; Supplementary Fig. S8) and higher CDAI remission attainment at 6 months (RR 1.80; 95% CI: 1.39, 2.33; *n* = 3 studies; Supplementary Fig. S9).

### Meta-regression of predictors of remission attainment

There was considerable between-study heterogeneity in pairwise meta-analyses of b/tsDMARDs plus methotrexate *vs* methotrexate (*I*² = 82%; τ² = 0.08) and b/tsDMARD monotherapy *vs* methotrexate (*I*² = 80%; τ² = 0.11). Random-effects meta-regression was performed to investigate whether study-level characteristics explained between-study heterogeneity in treatment effects for DAS28 remission attainment at the 6-month primary outcome timepoint. Studies with higher baseline DAS28 scores and a greater proportion of rheumatoid factor-positive participants were associated with larger improvements in remission rates for b/tsDMARDs plus methotrexate *vs* methotrexate alone (Fig. 2A; Supplementary Table S3; Supplementary Figs S10 and S11). Baseline DAS28 explained the greatest proportion of between-study heterogeneity, reducing residual heterogeneity to *I*² = 63% (τ² = 0.03; *R*² = 58%).

**Figure 2:**
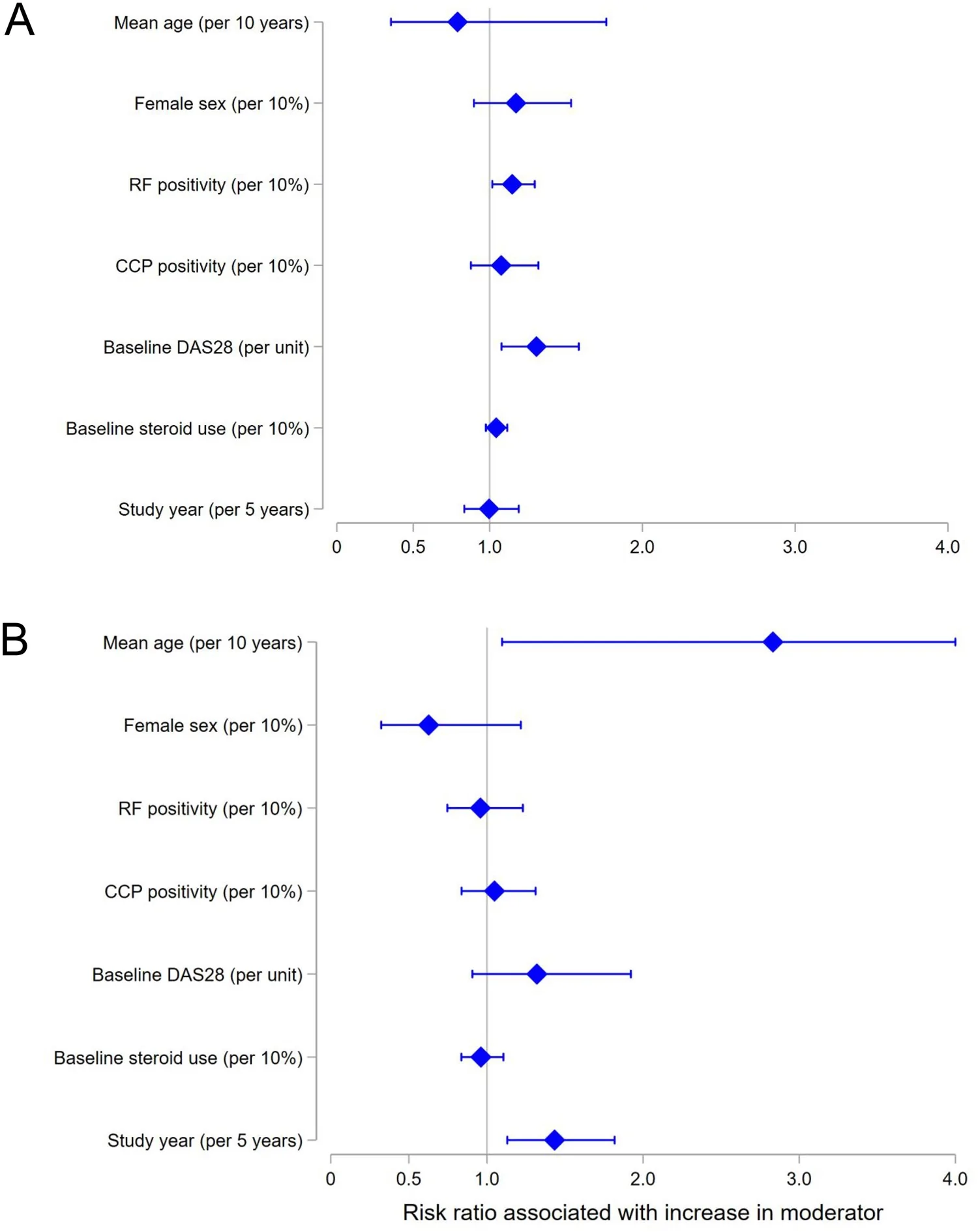
Pairwise meta-regression of predictors of treatment effect heterogeneity for DAS28 remission at 6 months, comparing b/tsDMARDs plus methotrexate *vs* methotrexate alone (**A**, top panel) and b/tsDMARD monotherapy *vs* methotrexate (**B**, bottom panel). Forest plots show risk ratios and 95% confidence intervals from random-effects meta-regression analyses of study-level characteristics. Values >1 indicate larger treatment effects with increasing values of the moderator. Confidence intervals extending beyond the plotting range are truncated. b/tsDMARDs: biologic and targeted synthetic DMARDs; CCP: anti-cyclic citrullinated peptide; DAS28: Disease Activity Score in 28 joints; RF: rheumatoid factor

In analyses of b/tsDMARD monotherapy *vs* methotrexate, larger improvements in remission rates were associated with higher mean participant age and more recent study year, although confidence intervals were wider (Fig. 2B; Supplementary Table S3; Supplementary Figs S12 and S13). Study year explained the greatest proportion of between-study heterogeneity, reducing residual heterogeneity to *I*² = 56% (τ² = 0.04; *R*² = 66%). Visual inspection of funnel plots suggested some asymmetry for both comparisons (Supplementary Fig. S14), although Egger’s test failed to reject the null hypothesis of small-study effects for b/tsDMARDs plus methotrexate *vs* methotrexate alone (*P* *=* 0.98) or b/tsDMARD monotherapy *vs* methotrexate (*P* *=* 0.095).

### Network meta-analysis

To investigate whether treatment effects differed between b/tsDMARD classes, network meta-analysis was performed to compare DAS28 remission outcomes across individual b/tsDMARD classes using direct and indirect evidence. At 6 months, all b/tsDMARD classes in combination with methotrexate demonstrated higher DAS28 remission rates than methotrexate alone: TNFi (RR 1.50; 95% CI: 1.24, 1.81), abatacept (RR 1.56; 95% CI: 1.15, 2.13), tocilizumab (RR 1.80; 95% CI: 1.32, 2.45) and JAKi (RR 1.83; 95% CI: 1.25, 2.66) (Fig. 3). Similar findings were observed at 3 and 12 months (Supplementary Fig. S15 and S16). There was evidence of global inconsistency (χ² = 13.00, *P* = 0.0046) between direct and indirect evidence. Node-splitting analyses demonstrated inconsistency for TNFi plus methotrexate *vs* methotrexate (*P* = 0.022) and abatacept plus methotrexate *vs* methotrexate (*P* = 0.047), but not for other comparisons. Between-study heterogeneity was similar in class-specific models (τ = 0.26) and models that combined b/tsDMARDs into one node (τ = 0.25), suggesting that differences between b/tsDMARD classes did not contribute substantially to the observed heterogeneity. Network meta-analyses comparing ACR50 attainment at 6 months showed comparable effect estimates for TNFi (RR 1.33; 95% CI: 1.17, 1.50), tocilizumab (RR 1.33; 95% CI: 1.02, 1.73), abatacept (RR 1.34; 95% CI: 0.98, 1.82), and JAKi (RR 1.48; 95% CI: 1.15, 1.89) (Supplementary Fig. S17), with no eligible rituximab studies. Network comparisons were not possible for CDAI remission due to insufficient numbers of studies reporting this outcome.

**Figure 3:**
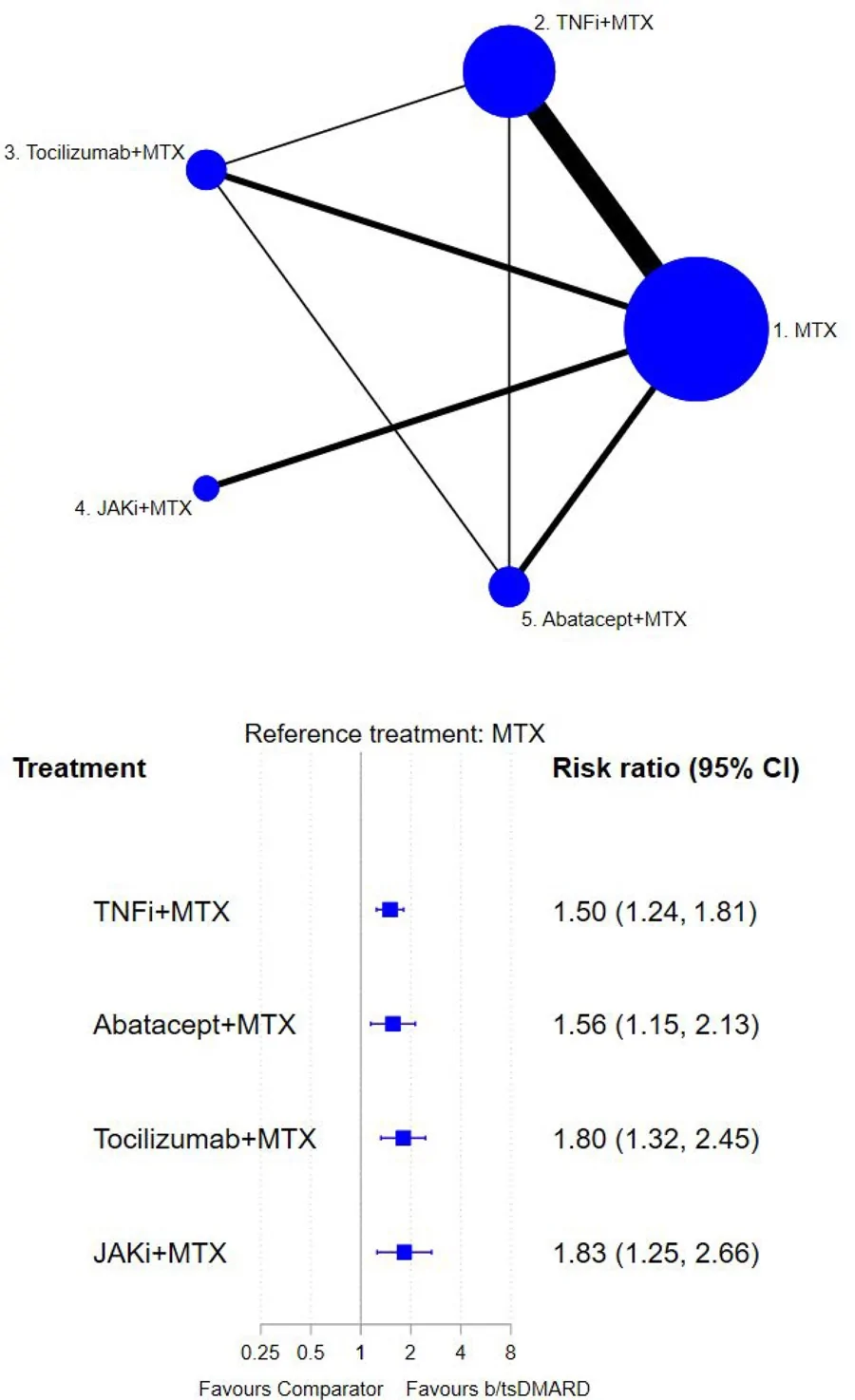
Network meta-analysis of DAS28 remission attainment at 6 months post-randomization, comparing b/tsDMARDs plus methotrexate *vs* methotrexate-based comparators in participants with early RA who had not previously been treated with methotrexate. A network map of treatment comparisons is shown in the top panel. A forest plot comparing risk ratios for DAS28 remission between treatment comparisons is shown in the bottom panel. b/tsDMARD: biologic and targeted synthetic DMARDs; DAS28: Disease Activity Score in 28 joints; JAKi: Janus kinase inhibitor; MTX: methotrexate; TNFi: TNF inhibitor

Network estimates for b/tsDMARD monotherapy were less precise because of the smaller number of eligible studies. At 6 months, tocilizumab (RR 2.10; 95% CI: 1.56, 2.81) and JAKi (RR 2.22; 95% CI: 1.74, 2.83) demonstrated higher DAS28 remission rates than methotrexate, whereas TNFi (RR 0.89; 95% CI: 0.54, 1.48) and abatacept (RR 1.23; 95% CI: 0.72, 2.08) monotherapy did not (Fig. 4). Similar findings were observed at 12 months, while at 3 months only tocilizumab demonstrated superior remission attainment (Supplementary Figs S18 and S19). No direct comparisons between b/tsDMARD classes were available within the monotherapy network, precluding formal assessment of inconsistency. Between-study heterogeneity was substantially lower in the class-specific monotherapy network than in a model combining all b/tsDMARD monotherapies (τ = 0.18 *vs* 0.34), suggesting that differences between b/tsDMARD classes accounted for a substantial proportion of the observed heterogeneity. Network comparisons were not possible for ACR50 response or CDAI remission due to smaller numbers of studies reporting these outcomes.

**Figure 4:**
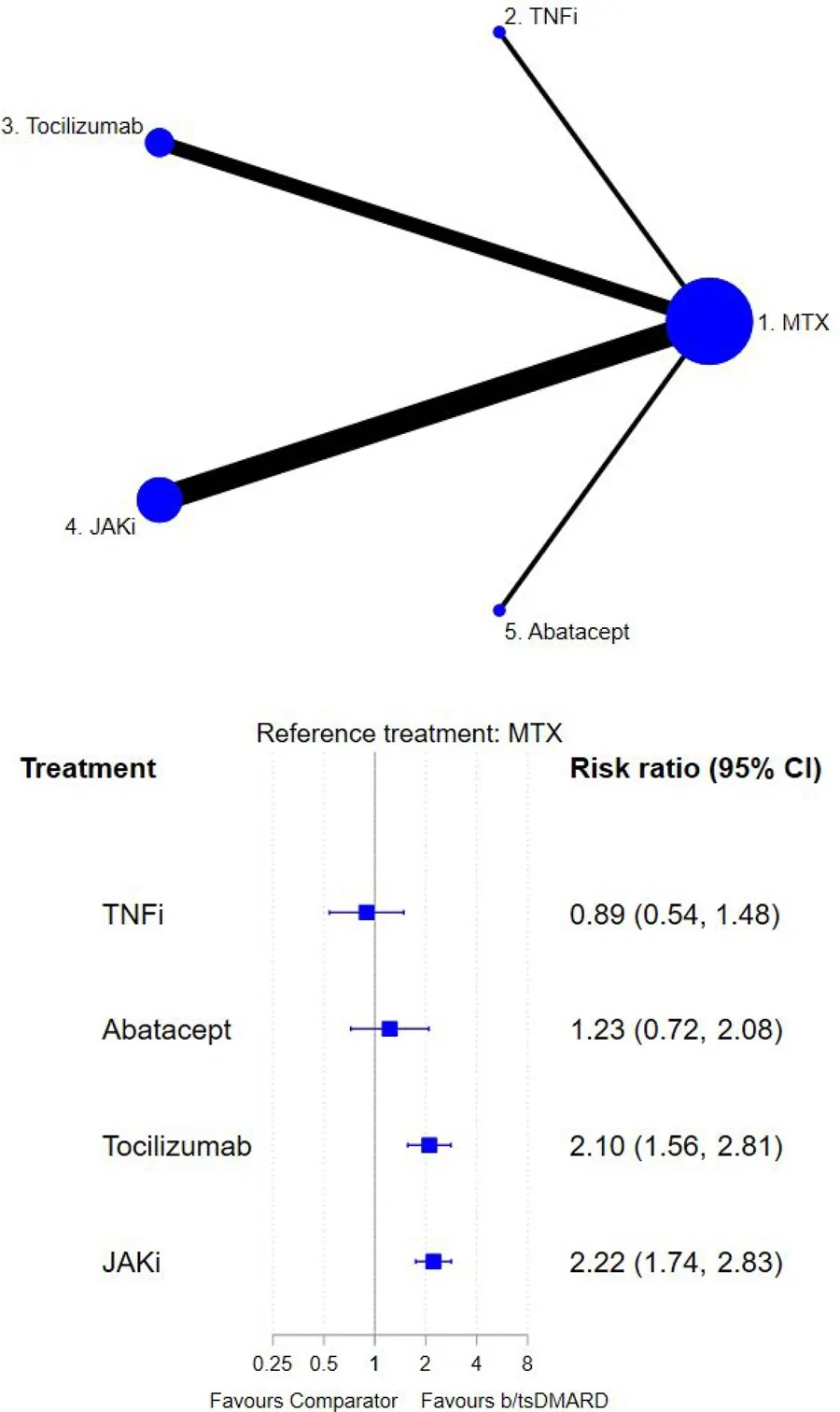
Network meta-analysis of DAS28 remission attainment at 6 months post-randomization, comparing b/tsDMARD monotherapy *vs* methotrexate in participants with early RA who had not previously been treated with methotrexate. A network map of treatment comparisons is shown in the top panel. A forest plot comparing risk ratios for DAS28 remission between treatment comparisons is shown in the bottom panel. b/tsDMARD: biologic and targeted synthetic DMARDs; DAS28: Disease Activity Score in 28 joints; JAKi: Janus kinase inhibitor; MTX: methotrexate; TNFi: TNF inhibitor

### Sensitivity analyses

Four studies included comparator arms in which participants were randomized to receive methotrexate in combination with other csDMARDs and/or corticosteroids, rather than methotrexate alone [18–21]. Sensitivity pairwise meta-analyses excluding these trials yielded results consistent with the primary analysis (sensitivity analysis: RR 1.80; 95% CI: 1.63, 1.98; *n* = 14 studies; primary analysis: RR 1.62; 95% CI: 1.39, 1.89; *n* = 17 studies; Supplementary Fig. S20). In leave-one-out meta-analyses, the sequential exclusion of individual studies did not substantially influence the pooled effect-estimates, indicating that the findings were not driven by any single study (Supplementary Fig. S21). In sensitivity analyses restricted to studies enrolling participants with no prior exposure to any csDMARDs, findings were consistent with the primary analysis (Supplementary Fig. S22). Similarly, analyses that excluded studies deemed at high risk of bias produced comparable estimates to the primary analysis (Supplementary Fig. S23).

## Discussion

In this meta-analysis of 29 RCTs with over 13 000 methotrexate-naïve participants with early RA, initial treatment with b/tsDMARDs plus methotrexate was associated with significantly higher remission rates compared with methotrexate alone. These benefits were observed for all b/tsDMARD classes from 3 months onwards. Meta-regression analyses indicated that the beneficial effects of b/tsDMARDs in combination with methotrexate were greater in studies enrolling participants with highly active disease and greater proportions of rheumatoid factor-positive participants. For b/tsDMARD monotherapy, pooled analyses also demonstrated higher remission rates compared with methotrexate alone, although class-specific benefits were limited to tocilizumab and JAKi.

Our findings are consistent with earlier meta-analyses of b/tsDMARD initiation in patients with recent-onset RA [22–25]. A previous Cochrane review, which included studies published up to 2015, reported significantly higher DAS28 remission rates with biologics plus methotrexate compared with csDMARD comparators in methotrexate-naïve participants with RA (*n* = 15 RCTs) [22]. However, data on biologic monotherapy were more limited at the time, with only two eligible RCTs in methotrexate-naïve patients and no evidence of significant benefit [22]. Since then, numerous additional trials of b/tsDMARDs in early RA have been published, including RCTs for baricitinib, upadacitinib and filgotinib, as well as head-to-head strategy trials such as NORD-STAR [18]. In our meta-analysis, we incorporated data on 29 RCTs, including 11 studies comparing b/tsDMARD monotherapy with methotrexate, enabling us to evaluate the benefits of early b/tsDMARD initiation across individual drug classes, both as combination therapy with methotrexate and as monotherapy. Benefits were present from 3 months onwards and were consistent across several sensitivity analyses, including analyses restricted to studies enrolling participants without any prior csDMARD exposure.

In network meta-analyses of b/tsDMARDs combined with methotrexate, all b/tsDMARD classes demonstrated superior remission rates compared with methotrexate alone, although some inconsistency between direct and indirect estimates was observed within the network. Between-study heterogeneity was similar whether b/tsDMARDs were analysed as a pooled group or separated into individual drug classes, suggesting that differences between b/tsDMARD classes did not substantially contribute to variation in treatment effects between studies. For b/tsDMARD monotherapy, the ability to draw inferences was more limited due to smaller numbers of eligible studies; however, evidence of benefit was observed for tocilizumab and JAKi, in line with previous studies demonstrating the efficacy of these classes as monotherapy [26, 27]. In contrast to analyses of b/tsDMARDs combined with methotrexate, between-study heterogeneity was substantially lower in class-specific models, suggesting that differences between b/tsDMARD monotherapy classes contributed to the heterogeneity in treatment effects between studies.

An important finding was that the benefit of b/tsDMARDs plus methotrexate was greatest early on, with patients 95% more likely to attain DAS28 remission at 3 months, compared with 54% more likely at 12 months. This suggests that a key advantage of initial b/tsDMARD therapy is more rapid disease control. The attenuation over time may reflect intensification of therapy in comparator groups, including treatment escalation, rescue therapy and crossover to b/tsDMARDs. A similar pattern has been observed in strategy trials such as TEAR, in which the early clinical advantage of upfront combination therapy diminished following treatment escalation in the step-up arms [28]. Conversely, studies including BeSt suggest that achieving disease control earlier may confer longer-term benefits for outcomes such as radiographic progression [11]. Importantly, the first months following an RA diagnosis represent a critical period during which active disease can contribute to impaired function, employment loss and poor psychological health [29, 30]. Our findings highlight the potential value of upfront therapy with b/tsDMARDs in achieving early remission, particularly in patients with poor prognostic factors.

Current RA treatment guidelines from EULAR, ACR and NICE recommend methotrexate as initial therapy for most patients with RA, including those with high disease activity at diagnosis [1, 31, 32]. In England and Wales, NICE guidance permits escalation to b/tsDMARDs only for RA patients who have responded inadequately to, or been intolerant of, at least two csDMARDs, a requirement that has been in place since 2002 [2]. In recently updated EULAR recommendations, escalation to b/tsDMARDs is recommended after failure to respond adequately to the initial csDMARD strategy; however, b/tsDMARDs were not recommended as first-line therapy for several reasons, including the absence of trial evidence demonstrating superiority of b/tsDMARDs over methotrexate plus corticosteroids in early RA (citing the NORD-STAR and IDEA studies) [18, 19], alongside safety considerations and higher acquisition costs compared with csDMARDs [1]. Our findings provide additional evidence to inform this discussion. In the NORD-STAR study, DAS28 remission attainment was high (>60% at 24 weeks) across all treatment arms, including with conventional treatment [18, 33]. It is important to note, though, that the conventional treatment arm in NORD-STAR comprised rapidly escalated methotrexate combined with substantial doses of oral prednisolone (20 mg per day initially, tapered to 5 mg per day by week 9 and discontinued by week 36), or triple csDMARD therapy with mandatory intra-articular glucocorticoid injections in up to four swollen joints at each visit. Similarly, the conventional treatment arm in the IDEA study included a 250 mg intravenous dose of methylprednisolone at treatment initiation [19]. While these aggressive conventional treatment approaches were highly effective within trial settings, implementing such intensive strategies consistently in routine clinical practice is more challenging. Indeed, National Clinical Audit data from England and Wales show that less intensive treat-to-target strategies are still not consistently delivered in routine care [34]. Moreover, follow-up data from NORD-STAR showed that the initial similarity between treatment arms became less apparent after corticosteroids were discontinued, with increasing separation between the b/tsDMARD and csDMARD groups [33, 35].

Safety and cost considerations have also evolved since many of the pivotal trials informing current treatment strategies were conducted. With respect to safety, meta-analyses in early RA have shown comparable adverse event rates between b/tsDMARDs and csDMARDs [24, 25]. Early disease control can also help to mitigate the long-term risks associated with persistent inflammation, including infection and malignancy [36, 37]. From a cost perspective, the acquisition costs of biosimilars in many countries are now substantially lower than those of originator biologics at the time of their introduction. As a result, the incremental treatment costs associated with earlier b/tsDMARD use may be offset by reductions in disease-related morbidity, disability and work impairment, particularly among individuals with poor prognostic factors at diagnosis [38, 39].

Our study has a number of strengths. We meta-analysed data from 29 RCTs and >13 000 participants to evaluate the efficacy of b/tsDMARDs plus methotrexate and b/tsDMARD monotherapy across multiple time-points. We showed consistent findings in several sensitivity analyses, and utilized network meta-analysis to explore treatment effects across different b/tsDMARD classes. Using meta-regression, we were able to demonstrate greater treatment effects with b/tsDMARDs plus methotrexate in studies enrolling participants with more active RA and higher proportions of seropositive participants, while b/tsDMARD monotherapy was associated with greater treatment benefits in more recently conducted studies and those enrolling participants with higher mean age.

It is also important to acknowledge several limitations. There was substantial between-study heterogeneity, reflecting differences in study populations, treatment protocols and outcome ascertainment. For example, there were notable differences in comparator csDMARD regimens, with some studies opting for rapidly escalating methotrexate doses combined with additional csDMARDs and/or steroids, while others adopted less intensive regimens. The pooled estimates therefore do not necessarily represent a uniform comparison between first-line b/tsDMARD therapy and an optimized methotrexate treat-to-target strategy, which should be considered when applying these findings to current clinical practice. We selected DAS28 remission as the primary outcome, as it is widely used to assess treatment response in clinical practice and trials. DAS28-CRP was prioritized where available to maximize consistency; however, some studies reported only DAS28-ESR. As these measures are not fully interchangeable, this may have introduced additional measurement heterogeneity. It is also important to note that DAS28 is strongly influenced by levels of acute phase reactants (e.g. CRP), which in turn can be differentially impacted across b/tsDMARD classes. This may have contributed to the benefits observed for JAKi and tocilizumab in monotherapy analyses, which are known to preferentially reduce acute phase reactants. Indeed, in contrast to analyses of b/tsDMARDs plus methotrexate, between-study heterogeneity was substantially lower in class-specific monotherapy analyses than when b/tsDMARD monotherapies were pooled into a single node, suggesting that differences between b/tsDMARD monotherapy classes accounted for a substantial proportion of the observed heterogeneity. To try and mitigate this, we included ACR50 response and CDAI remission as secondary outcomes, which are less influenced by acute phase reactants than DAS28; however, fewer studies reported these outcomes, particularly for CDAI remission, resulting in less precise estimates.

Similarly, we opted not to present network-derived estimates for head-to-head comparisons between b/tsDMARD classes, as these would have relied predominantly on indirect evidence and therefore been associated with considerable uncertainty. Indirect comparisons between b/tsDMARD classes should be interpreted cautiously, as older trials (e.g. those evaluating TNFi) and more recent studies (e.g. those evaluating JAKi) may have enrolled different populations, reflecting changes in trial design and clinical practice over time, which could challenge the transitivity assumption required for valid indirect comparisons. In addition, evidence of inconsistency was identified for some network comparisons, indicating discrepancies between direct and indirect estimates and adding uncertainty to the network estimates. While meta-regression was used to explore study-level predictors of treatment effect heterogeneity, our analyses were based on aggregated study-level data rather than individual participant data, which limited our ability to identify patient-level predictors of response. These findings were derived from trials designed to evaluate efficacy under controlled conditions, which may limit their generalizability to routine clinical practice. Our analyses were restricted to efficacy outcomes during the first year of treatment and did not assess safety, radiographic progression, functional improvement, cost-effectiveness or polypharmacy considerations. These factors are important when considering earlier use of b/tsDMARDs, particularly for treatments such as JAKi, for which treatment selection is influenced by individual risk factors.

In conclusion, in methotrexate-naïve patients with early RA, initial treatment with b/tsDMARDs plus methotrexate was associated with consistently higher remission rates than methotrexate alone across multiple timepoints. As monotherapy, JAKi and tocilizumab had superior remission rates to methotrexate. These findings suggest that a uniform methotrexate-first approach may not be optimal for all patients with early RA. They support further evaluation of earlier b/tsDMARD initiation and suggest that treatment effects may differ according to baseline disease characteristics, while longer-term outcomes, safety and cost-effectiveness should also be considered.

## Supplementary Material

keag515_Supplementary_Data

## Data Availability

All data utilized in this study are derived from publicly available sources. Aggregated, study-level data extracted from these sources are available from the authors on request.
