# Supplementary material for: First-line biologic and targeted synthetic DMARDs versus methotrexate in early rheumatoid arthritis: a meta-analysis of remission outcomes
Source: Rheumatology (Oxford). 2026 Sep 17;65(10):keag515. doi: 10.1093/rheumatology/keag515 (PMC13645451; doi:10.1093/rheumatology/keag515)
Supplement: Supplementary file 1 [file keag515_supplementary_data.docx]

**Supplementary Figures and Tables**

[**Supplementary Figure S1*.*** PRISMA flowchart of study selection.	3](#_Toc237514807)

[**Supplementary Figure S2*.*** Pairwise meta-analysis of DAS28 remission attainment at 3 months for b/tsDMARDs plus methotrexate vs. methotrexate alone.	4](#_Toc237514808)

[**Supplementary Figure S3*.*** Pairwise meta-analysis of DAS28 remission attainment at 12 months for b/tsDMARDs plus methotrexate vs. methotrexate alone.	5](#_Toc237514809)

[**Supplementary Figure S4*.*** Pairwise meta-analysis of ACR50 attainment at 6 months for b/tsDMARDs plus methotrexate vs. methotrexate alone.	6](#_Toc237514810)

[**Supplementary Figure S5*.*** Pairwise meta-analysis of CDAI remission attainment at 6 months for b/tsDMARDs plus methotrexate vs. methotrexate alone.	7](#_Toc237514811)

[**Supplementary Figure S6*.*** Pairwise meta-analysis of DAS28 remission attainment at 3 months for b/tsDMARD monotherapy vs. methotrexate.	8](#_Toc237514812)

[**Supplementary Figure S7*.*** Pairwise meta-analysis of DAS28 remission attainment at 12 months for b/tsDMARD monotherapy vs. methotrexate.	9](#_Toc237514813)

[**Supplementary Figure S8*.*** Pairwise meta-analysis of ACR50 attainment at 6 months for b/tsDMARD monotherapy vs. methotrexate.	10](#_Toc237514814)

[**Supplementary Figure S9*.*** Pairwise meta-analysis of CDAI remission attainment at 6 months for b/tsDMARD monotherapy vs. methotrexate.	11](#_Toc237514815)

[**Supplementary Figure S10*.*** Bubble plot of the association between mean baseline DAS28 and treatment effect on DAS28 remission at 6 months for b/tsDMARDs plus methotrexate vs. methotrexate-based comparators.	12](#_Toc237514816)

[**Supplementary Figure S11*.*** Bubble plot of the association between rheumatoid factor positivity and treatment effect on DAS28 remission at 6 months for b/tsDMARDs plus methotrexate vs. methotrexate-based comparators.	13](#_Toc237514817)

[**Supplementary Figure S12*.*** Bubble plot of the association between mean participant age and treatment effect on DAS28 remission at 6 months for b/tsDMARD monotherapy vs. methotrexate.	14](#_Toc237514818)

[**Supplementary Figure S13*.*** Bubble plot of the association between year of study publication and treatment effect on DAS28 remission at 6 months for b/tsDMARD monotherapy vs. methotrexate.	15](#_Toc237514819)

[**Supplementary Figure S14*.*** Funnel plots assessing small-study effects for DAS28 remission at 6 months.	16](#_Toc237514820)

[**Supplementary Figure S15*.*** Network meta-analysis of DAS28 remission attainment at 3 months for classes of b/tsDMARD plus methotrexate vs. methotrexate.	17](#_Toc237514821)

[**Supplementary Figure S16*.*** Network meta-analysis of DAS28 remission attainment at 12 months for classes of b/tsDMARD plus methotrexate vs. methotrexate.	18](#_Toc237514822)

[**Supplementary Figure S17*.*** Network meta-analysis of ACR50 attainment at 6 months for classes of b/tsDMARD plus methotrexate vs. methotrexate.	19](#_Toc237514823)

[**Supplementary Figure S18*.*** Network meta-analysis of DAS28 remission attainment at 3 months for classes of b/tsDMARD monotherapy vs. methotrexate.	20](#_Toc237514824)

[**Supplementary Figure S19*.*** Network meta-analysis of DAS28 remission attainment at 12 months for classes of b/tsDMARD monotherapy vs. methotrexate.	21](#_Toc237514825)

[**Supplementary Figure S20.** Sensitivity analysis of DAS28 remission at 6 months after exclusion of trials with combination comparator regimens.	22](#_Toc237514826)

[**Supplementary Figure S21**. Leave-one-out sensitivity analyses of DAS28 remission at 6 months.	23](#_Toc237514827)

[**Supplementary Figure S22**. Sensitivity analysis of DAS28 remission at 6 months, restricted to participants with no prior exposure to csDMARDs.	24](#_Toc237514828)

[**Supplementary Figure S23**. Sensitivity analysis of DAS28 remission at 6 months, excluding studies deemed at high risk of bias.	25](#_Toc237514829)

[**Supplementary Table S1.** Details of included studies.	26](#_Toc237514830)

[**Supplementary Table S2.** Risk of bias assessment for included studies.	29](#_Toc237514831)

[**Supplementary Table S3.** Study-level moderators of treatment effect heterogeneity from pairwise meta-regression of DAS28 remission attainment at 6 months.	31](#_Toc237514832)

[**Supplementary Data.** Database search strategies.	32](#_Toc237514833)

[**References**	33](#_Toc237514834)

## **Supplementary Figure S1*.*** PRISMA flowchart of study selection.

## **Supplementary Figure S2*.*** Pairwise meta-analysis of DAS28 remission attainment at 3 months for b/tsDMARDs plus methotrexate vs. methotrexate alone.

DAS28 remission attainment at 3 months post-randomisation was compared using pairwise meta-analysis for b/tsDMARDs plus methotrexate vs. methotrexate-based comparators in participants with early RA who had not previously been treated with methotrexate. b/tsDMARD: biologic and targeted synthetic DMARDs; CI: confidence interval.

## **Supplementary Figure S3*.*** Pairwise meta-analysis of DAS28 remission attainment at 12 months for b/tsDMARDs plus methotrexate vs. methotrexate alone.

DAS28 remission attainment at 12 months post-randomisation was compared using pairwise meta-analysis for b/tsDMARDs plus methotrexate vs. methotrexate-based comparators in participants with early RA who had not previously been treated with methotrexate. b/tsDMARD: biologic and targeted synthetic DMARDs; CI: confidence interval.

## **Supplementary Figure S4*.*** Pairwise meta-analysis of ACR50 attainment at 6 months for b/tsDMARDs plus methotrexate vs. methotrexate alone.

American College of Rheumatology 50% response criteria (ACR50) attainment at 6 months post-randomisation was compared using pairwise meta-analysis for b/tsDMARDs plus methotrexate vs. methotrexate-based comparators in participants with early RA who had not previously been treated with methotrexate. b/tsDMARD: biologic and targeted synthetic DMARDs; CI: confidence interval.

## **Supplementary Figure S5*.*** Pairwise meta-analysis of CDAI remission attainment at 6 months for b/tsDMARDs plus methotrexate vs. methotrexate alone.

Clinical Disease Activity Index (CDAI) remission attainment at 6 months post-randomisation was compared using pairwise meta-analysis for b/tsDMARDs plus methotrexate vs. methotrexate-based comparators in participants with early RA who had not previously been treated with methotrexate. b/tsDMARD: biologic and targeted synthetic DMARDs; CI: confidence interval.

## **Supplementary Figure S6*.*** Pairwise meta-analysis of DAS28 remission attainment at 3 months for b/tsDMARD monotherapy vs. methotrexate.

DAS28 remission attainment at 3 months post-randomisation was compared using pairwise meta-analysis for b/tsDMARD monotherapy vs. methotrexate-based comparators in participants with early RA who had not previously been treated with methotrexate. b/tsDMARD: biologic and targeted synthetic DMARDs; CI: confidence interval.

## **Supplementary Figure S7*.*** Pairwise meta-analysis of DAS28 remission attainment at 12 months for b/tsDMARD monotherapy vs. methotrexate.

DAS28 remission attainment at 12 months post-randomisation was compared using pairwise meta-analysis for b/tsDMARD monotherapy vs. methotrexate-based comparators in participants with early RA who had not previously been treated with methotrexate. b/tsDMARD: biologic and targeted synthetic DMARDs; CI: confidence interval.

## **Supplementary Figure S8*.*** Pairwise meta-analysis of ACR50 attainment at 6 months for b/tsDMARD monotherapy vs. methotrexate.

American College of Rheumatology 50% response criteria (ACR50) attainment at 6 months post-randomisation was compared using pairwise meta-analysis for b/tsDMARD monotherapy vs. methotrexate-based comparators in participants with early RA who had not previously been treated with methotrexate. b/tsDMARD: biologic and targeted synthetic DMARDs; CI: confidence interval.

## **Supplementary Figure S9*.*** Pairwise meta-analysis of CDAI remission attainment at 6 months for b/tsDMARD monotherapy vs. methotrexate.

Clinical Disease Activity Index (CDAI) remission attainment at 6 months post-randomisation was compared using pairwise meta-analysis for b/tsDMARD monotherapy vs. methotrexate-based comparators in participants with early RA who had not previously been treated with methotrexate. b/tsDMARD: biologic and targeted synthetic DMARDs; CI: confidence interval.

## **Supplementary Figure S10*.*** Bubble plot of the association between mean baseline DAS28 and treatment effect on DAS28 remission at 6 months for b/tsDMARDs plus methotrexate vs. methotrexate-based comparators.

Bubble plot showing the association between mean baseline DAS28 and treatment effect of b/tsDMARDs plus methotrexate vs. methotrexate-based comparators on DAS28 remission at 6 months post-randomisation. Each circle represents a study, with circle size proportional to its random-effects meta-regression weight. The solid line represents the fitted meta-regression relationship, and the shaded area represents the 95% confidence interval (CI).

## **Supplementary Figure S11*.*** Bubble plot of the association between rheumatoid factor positivity and treatment effect on DAS28 remission at 6 months for b/tsDMARDs plus methotrexate vs. methotrexate-based comparators.

Bubble plot showing the association between the percentage of participants with rheumatoid factor (RF) positivity and treatment effect of b/tsDMARDs plus methotrexate vs. methotrexate-based comparators on DAS28 remission at 6 months post-randomisation. Each circle represents a study, with circle size proportional to its random-effects meta-regression weight. The solid line represents the fitted meta-regression relationship, and the shaded area represents the 95% confidence interval (CI).

## **Supplementary Figure S12*.*** Bubble plot of the association between mean participant age and treatment effect on DAS28 remission at 6 months for b/tsDMARD monotherapy vs. methotrexate.

Bubble plot showing the association between mean participant age and treatment effect of b/tsDMARD monotherapy vs. methotrexate on DAS28 remission at 6 months post-randomisation. Each circle represents a study, with circle size proportional to its random-effects meta-regression weight. The solid line represents the fitted meta-regression relationship, and the shaded area represents the 95% confidence interval (CI).

## **Supplementary Figure S13*.*** Bubble plot of the association between year of study publication and treatment effect on DAS28 remission at 6 months for b/tsDMARD monotherapy vs. methotrexate.

Bubble plot showing the association between the year of study publication and treatment effect of b/tsDMARD monotherapy vs. methotrexate on DAS28 remission at 6 months post-randomisation. Each circle represents a study, with circle size proportional to its random-effects meta-regression weight. The solid line represents the fitted meta-regression relationship, and the shaded area represents the 95% confidence interval (CI).

## **Supplementary Figure S14*.*** Funnel plots assessing small-study effects for DAS28 remission at 6 months.

A

B

Funnel plots for studies reporting DAS28 remission at 6 months post-randomisation. Panel A shows b/tsDMARDs plus methotrexate vs. methotrexate-based comparators, and Panel B shows b/tsDMARD monotherapy vs. methotrexate-based comparators. The vertical line represents the pooled effect estimate, and diagonal lines indicate pseudo 95% confidence intervals (CI).

## **Supplementary Figure S15*.*** Network meta-analysis of DAS28 remission attainment at 3 months for classes of b/tsDMARD plus methotrexate vs. methotrexate.

DAS28 remission attainment at 3 months post-randomisation was compared using network meta-analysis between classes of b/tsDMARD plus methotrexate, vs. methotrexate alone, in participants with early RA who had not previously been treated with methotrexate. A network map of treatment comparisons is shown in the top panel. A forest plot comparing risk ratios for DAS28 remission between treatment comparisons is shown in the bottom panel. b/tsDMARD: biologic and targeted synthetic DMARDs; CI: confidence interval; JAKi: Janus kinase inhibitor; MTX: methotrexate; TNFi: TNF inhibitor.

## **Supplementary Figure S16*.*** Network meta-analysis of DAS28 remission attainment at 12 months for classes of b/tsDMARD plus methotrexate vs. methotrexate.

DAS28 remission attainment at 12 months post-randomisation was compared using network meta-analysis between classes of b/tsDMARD plus methotrexate vs. methotrexate alone in participants with early RA who had not previously been treated with methotrexate. A network map of treatment comparisons is shown in the top panel. A forest plot comparing risk ratios for DAS28 remission between treatment comparisons is shown in the bottom panel. b/tsDMARD: biologic and targeted synthetic DMARDs; CI: confidence interval; JAKi: Janus kinase inhibitor; MTX: methotrexate; TNFi: TNF inhibitor.

## **Supplementary Figure S17*.*** Network meta-analysis of ACR50 attainment at 6 months for classes of b/tsDMARD plus methotrexate vs. methotrexate.

American College of Rheumatology 50% response criteria (ACR50) attainment at 6 months post-randomisation was compared using network meta-analysis between classes of b/tsDMARD plus methotrexate vs. methotrexate alone in participants with early RA who had not previously been treated with methotrexate. A network map of treatment comparisons is shown in the top panel. A forest plot comparing risk ratios for ACR50 response between treatment comparisons is shown in the bottom panel. b/tsDMARD: biologic and targeted synthetic DMARDs; CI: confidence interval; JAKi: Janus kinase inhibitor; MTX: methotrexate; TNFi: TNF inhibitor.

## **Supplementary Figure S18*.*** Network meta-analysis of DAS28 remission attainment at 3 months for classes of b/tsDMARD monotherapy vs. methotrexate.

DAS28 remission attainment at 3 months post-randomisation was compared using network meta-analysis between classes of b/tsDMARD monotherapy vs. methotrexate alone in participants with early RA who had not previously been treated with methotrexate. A network map of treatment comparisons is shown in the top panel. A forest plot comparing risk ratios for DAS28 remission between treatment comparisons is shown in the bottom panel. b/tsDMARD: biologic and targeted synthetic DMARDs; CI: confidence interval; JAKi: Janus kinase inhibitor; MTX: methotrexate; TNFi: TNF inhibitor.

## **Supplementary Figure S19*.*** Network meta-analysis of DAS28 remission attainment at 12 months for classes of b/tsDMARD monotherapy vs. methotrexate.

DAS28 remission attainment at 12 months post-randomisation was compared using network meta-analysis between classes of b/tsDMARD monotherapy vs. methotrexate alone in participants with early RA who had not previously been treated with methotrexate. A network map of treatment comparisons is shown in the top panel. A forest plot comparing risk ratios for DAS28 remission between treatment comparisons is shown in the bottom panel. b/tsDMARD: biologic and targeted synthetic DMARDs; CI: confidence interval; JAKi: Janus kinase inhibitor; MTX: methotrexate; TNFi: TNF inhibitor.

## **Supplementary Figure S20.** Sensitivity analysis of DAS28 remission at 6 months after exclusion of trials with combination comparator regimens.

DAS28 remission attainment at 6 months post-randomisation was compared using pairwise meta-analysis for b/tsDMARDs plus methotrexate vs. methotrexate-based comparators. Trials in which comparator regimens included methotrexate in combination with other csDMARDs and/or corticosteroids were excluded. Comparable sensitivity meta-analyses are not presented for b/tsDMARD monotherapy vs. methotrexate because no studies included comparator regimens comprising methotrexate in combination with other csDMARDs and/or corticosteroids. b/tsDMARD: biologic and targeted synthetic DMARDs; CI: confidence interval; csDMARD: conventional synthetic disease-modifying antirheumatic drug.

## **Supplementary Figure S21**. Leave-one-out sensitivity analyses of DAS28 remission at 6 months.

A

B

Leave-one-out sensitivity pairwise meta-analyses of DAS28 remission attainment at 6 months post-randomisation. Each row represents the pooled risk ratio obtained from pairwise meta-analyses after excluding the indicated study. Panel A shows b/tsDMARDs plus methotrexate vs. methotrexate alone; Panel B shows b/tsDMARD monotherapy vs. methotrexate. b/tsDMARD: biologic and targeted synthetic DMARDs; CI: confidence interval.

## **Supplementary Figure S22**. Sensitivity analysis of DAS28 remission at 6 months, restricted to participants with no prior exposure to csDMARDs.

B

A

DAS28 remission attainment at 6 months post-randomisation was compared using pairwise meta-analysis in studies restricted to participants with no prior exposure to DMARDs. Panel A shows b/tsDMARDs plus methotrexate vs. methotrexate alone, and Panel B shows b/tsDMARD monotherapy vs. methotrexate. b/tsDMARD: biologic and targeted synthetic DMARDs; CI: confidence interval; csDMARD: conventional synthetic disease-modifying antirheumatic drug.

## **Supplementary Figure S23**. Sensitivity analysis of DAS28 remission at 6 months, excluding studies deemed at high risk of bias.

DAS28 remission attainment at 6 months post-randomisation was compared using pairwise meta-analysis excluding studies deemed to be at high risk of bias (n=2 studies were excluded for this analysis). No studies comparing DAS28 remission attainment at 6 months for b/tsDMARD monotherapy vs. methotrexate were deemed at high risk of bias. b/tsDMARD: biologic and targeted synthetic DMARDs; CI: confidence interval; csDMARD: conventional synthetic disease-modifying antirheumatic drug.

## **Supplementary Table S1.** Details of included studies.

| **Study Acronym** | **Year** | **b/tsDMARD**  **arm(s)** | **Comparator arm(s)** | **Naïve to all csDMARDs** | **Total participant**  **number** | **Mean age, years** | **Female participants, %** | **Mean baseline DAS28** | **Rheumatoid factor positive participants, %** | **CCP positive participants, %** | **Participants on baseline steroids, %** |
| --- | --- | --- | --- | --- | --- | --- | --- | --- | --- | --- | --- |
| **AGREE^1^** | 2009 | Abatacept + MTX | MTX | No | 509 | 49.9 | 77.6 | 6.3 | 96.4 | 89.0 | 35.6 |
| **ASPIRE^2^** | 2004 | Infliximab + MTX | MTX | No | 641 | 50.6 | 72.8 | 6.6^†^ | 71.0 | NS | 37.4 |
| **AVERT^3^** | 2019 | Abatacept  Abatacept + MTX | MTX | No | 351 | 47.0 | 77.8 | 5.4 | 95.2 | NS | 31.9 |
| **AVERT 2^4,5^** | 2023 | Abatacept + MTX | MTX | Yes | 752 | 49.0 | 78.7 | 5.6 | 92.9 | NS | 43.6 |
| **C-EARLY^6^** | 2017 | Certolizumab + MTX | MTX | Yes | 868 | 50.6 | 76.9 | 6.7^†^ | 96.8 | 83.9 | 32.7 |
| **C-OPERA^7^** | 2016 | Certolizumab + MTX | MTX | No | 316 | 49.2 | 81.0 | 5.4^†^ | 94.6 | 100 | 18.0 |
| **CIERA^8^** | 2007 | Infliximab + MTX | MTX | No | 29 | 51.8 | 68.9 | 5.3 | 65.6 | 58.0 | 0 |
| **COMET^9^** | 2008 | Etanercept + MTX | MTX | No | 528 | 51.4 | 73.5 | 6.5 | NS | 68.5 | 49.5 |
| **Conaghan *et al.* ^10^** | 2016 | Tofacitinib  Tofacitinib + MTX | MTX | No | 109 | 48.8 | 82.6 | 6.4^†^ | 75.5 | 76.5 | 52.3 |
| **Ducreux *et al.* ^11^** | 2013 | Tocilizumab | MTX | Yes | 30 | 52.7 | 76.7 | 4.5 | 79.3 | 66.1 | 10.0 |
| **EMPIRE^12^** | 2014 | Etanercept + MTX | MTX | Yes | 110 | 48.1 | 76.3 | 4.1 | 56.0 | 76.5 | 0 |
| **FINCH 3^13^** | 2021 | Filgotinib  Filgotinib + MTX | MTX | No | 1042 | 52.8 | 77.0 | 5.7 | NS | NS | 38.8 |
| **FUNCTION^14^** | 2016 | Tocilizumab  Tocilizumab + MTX | MTX | No | 869 | 49.7 | 78.0 | 6.7^†^ | 90.0 | 86.3 | 37.0 |
| **GO BEFORE^15,16^** | 2009 | Golimumab  Golimumab + MTX | MTX | No | 637 | 49.5 | 82.9 | 5.1 | NS | NS | 66.7 |
| **GUEPARD^17^** | 2009 | Adalimumab + MTX | MTX | Yes | 65 | 47.8 | 80.0 | 5.8^†^ | 73.6 | 72.6 | 30.8 |
| **HIT-HARD^18^** | 2013 | Adalimumab + MTX | MTX | Yes | 172 | 49.8 | 68.6 | 6.2^†^ | 66.3 | 52.9 | NS |
| **HOPEFUL 1^19,20^** | 2014 | Adalimumab + MTX | MTX | No | 334 | 54.0 | 81.4 | 5.8 | 84.4 | 84.1 | 32.0 |
| **IDEA Study^21^** | 2013 | Infliximab + MTX | MTX + IV methylpred | Yes | 112 | 53.3 | 68.8 | NS | 55.0 | 69.6 | 0 |
| **IMAGE^22^** | 2011 | Rituximab + MTX  + IV methylpred | MTX + IV methylpred | No | 748 | 48.0 | 81.3 | 7.1^†^ | 86.3 | NS | 46.3 |
| **JOBS Study^23^** | 2008 | Adalimumab + MTX | MTX | No | 148 | 47.0 | 55.9 | 5.9 | 95.5 | 63.5 | NS |
| **NORD-STAR^24,25^** | 2020 | Abatacept + MTX  Certolizumab + MTX  Tocilizumab + MTX | MTX + prednisolone or SSZ + HCQ | Yes | 795 | 54.3 | 69.3 | 5.0 | 74.8 | 82.3 | NS |
| **OPERA^26^** | 2014 | Adalimumab + MTX | MTX | Yes | 180 | 55.2 | 66.0 | 5.6 | 72.0 | 65.1 | 0 |
| **OPTIMA^27^** | 2014 | Adalimumab + MTX | MTX | No | 1032 | 50.5 | 74.0 | 6.0 | 88.0 | 84.0 | 43.5 |
| **ORAL Start^28^** | 2014 | Tofacitinib | MTX | No | 559 | 49.8 | 77.1 | 6.6^†^ | 83.0 | 85.5 | 48.6 |
| **PREMIER^29,30^** | 2005 | Adalimumab  Adalimumab + MTX | MTX | No | 799 | 52.0 | 74.5 | 6.3 | NS | NS | 35.9 |
| **RA-BEGIN^31^** | 2017 | Baricitinib  Baricitinib + MTX | MTX | Yes | 584 | 50.3 | 72.7 | 5.9 | 96.3 | 90.1 | 35.5 |
| **SELECT-EARLY^32,33^** | 2020 | Upadacitinib | MTX | No | 631 | 52.3 | 76.2 | 5.9 | NS | NS | 49.1 |
| **U-ACT-EARLY^34^** | 2016 | Tocilizumab  Tocilizumab + MTX | MTX | Yes | 317 | 53.8 | 66.9 | 5.2^†^ | 72.4 | 70.4 | 0 |
| **VEDERA^35^** | 2020 | Etanercept + MTX  + IM depomedrone | MTX + IM depomedrone | Yes | 120 | 50.0 | 71.0 | 5.7^†^ | 71.5 | 83.0 | 2.5 |

Eligible studies were randomised controlled trials comparing b/tsDMARDs, in combination with methotrexate or as monotherapy, against a methotrexate-based comparator in adults with early RA. Eligible trials were required to report remission outcomes using the Disease Activity Score in 28 joints (DAS28) at one or more pre-defined follow-up time points (3, 6 or 12 months post-randomisation). Where both DAS28-CRP and DAS28-ESR remission data were reported, DAS28-CRP was prioritised; DAS28-ESR data were used when CRP-based outcomes were not reported (denoted by a †). b/tsDMARD: biologic and targeted synthetic DMARDs; csDMARD: conventional synthetic disease-modifying antirheumatic drug; HCQ: hydroxychloroquine; IM: intramuscular; IV: intravenous; MTX: methotrexate; NS: not specified; SSZ: sulfasalazine.

## **Supplementary Table S2.** Risk of bias assessment for included studies.

| **Study name (year)** | **Bias from randomisation process (D1)** | **Bias due to deviations from intended interventions (D2)** | **Bias due to missing outcome data (D3)** | **Bias in measurement of outcome (D4)** | **Bias in selection of reported result (D5)** | **Overall** |
| --- | --- | --- | --- | --- | --- | --- |
| AGREE (2009) |  |  |  |  |  |  |
| ASPIRE (2004) |  |  |  |  |  |  |
| AVERT (2019) |  |  |  |  |  |  |
| AVERT 2 (2023) |  |  |  |  |  |  |
| C-EARLY (2017) |  |  |  |  |  |  |
| C-OPERA (2016) |  |  |  |  |  |  |
| CIERA (2007) |  |  |  |  |  |  |
| COMET (2008) |  |  |  |  |  |  |
| Conaghan *et al.* (2016) |  |  |  |  |  |  |
| Ducreux *et al.* (2013) |  |  |  |  |  |  |
| EMPIRE (2014) |  |  |  |  |  |  |
| FINCH 3 (2021) |  |  |  |  |  |  |
| FUNCTION (2016) |  |  |  |  |  |  |
| GO BEFORE (2009) |  |  |  |  |  |  |
| GUEPARD (2009) |  |  |  |  |  |  |
| HIT-HARD (2013) |  |  |  |  |  |  |
| HOPEFUL 1 (2014) |  |  |  |  |  |  |
| IDEA Study (2013) |  |  |  |  |  |  |
| IMAGE (2011) |  |  |  |  |  |  |
| JOBS Study (2008) |  |  |  |  |  |  |
| NORD-STAR (2020) |  |  |  |  |  |  |
| OPERA (2014) |  |  |  |  |  |  |
| OPTIMA (2013) |  |  |  |  |  |  |
| ORAL Start (2014) |  |  |  |  |  |  |
| PREMIER (2005) |  |  |  |  |  |  |
| RA-BEGIN (2017) |  |  |  |  |  |  |
| SELECT-EARLY (2020) |  |  |  |  |  |  |
| U-ACT-EARLY (2016) |  |  |  |  |  |  |
| VEDERA (2020) |  |  |  |  |  |  |

Risk of bias was assessed using the Cochrane Risk of Bias 2 (RoB 2) tool across five domains (D1-D5), with each domain judged as low risk (green), some concerns (yellow), or high risk of bias (red). Bias due to missing outcome data was assessed according to the extent and likely reasons for missingness and its potential impact on the effect estimate. Bias in selection of the reported result was assessed against pre-specified analyses in trial protocols or registrations, where available. Overall risk of bias was judged low if all domains were rated low risk, some concerns if at least one domain raised some concerns and no domain was rated high risk, and high risk if at least one domain was rated high risk.

## **Supplementary Table S3.** Study-level moderators of treatment effect heterogeneity from pairwise meta-regression of DAS28 remission attainment at 6 months.

| **Moderator (per unit increase)** | **Studies, n** | **Ratio of RRs**  **(95% CI)** | ***P-*value** | **Residual I²** | **Residual**  **τ²** | **R²** |
| --- | --- | --- | --- | --- | --- | --- |
| ***b/tsDMARDs plus MTX vs. MTX*** |  |  |  |  |  |  |
| Mean age, per 10 years | 17 | 0.79 (0.35–1.76) | 0.57 | 81.7% | 0.082 | 0.0% |
| RF positivity, per 10% | 14 | 1.15 (1.02–1.30) | 0.025 | 77.3% | 0.067 | 34.0% |
| CCP positivity, per 10% | 12 | 1.08 (0.88–1.32) | 0.48 | 87.2% | 0.13 | 0.0% |
| Baseline oral steroid use, per 10% | 15 | 1.04 (0.98–1.12) | 0.22 | 65.8% | 0.040 | 0.0% |
| Study year, per 5 years | 17 | 1.00 (0.83–1.19) | 0.97 | 82.3% | 0.085 | 0.0% |
| Female, per 10% | 17 | 1.17 (0.90–1.53) | 0.24 | 79.4% | 0.071 | 10.9% |
| Baseline DAS28, per 1 unit | 16 | 1.31 (1.08–1.58) | 0.007 | 62.8% | 0.030 | 58.3% |
|  |  |  |  |  |  |  |
| ***b/tsDMARD monotherapy vs. MTX*** |  |  |  |  |  |  |
| Mean age, per 10 years | 10 | 2.83 (1.10–7.31) | 0.031 | 71.6% | 0.069 | 36.1% |
| RF positivity, per 10% | 7 | 0.96 (0.75–1.23) | 0.74 | 48.1% | 0.046 | 0.0% |
| CCP positivity, per 10% | 6 | 1.05 (0.84–1.31) | 0.68 | 24.1% | 0.016 | 0.0% |
| Baseline oral steroid use, per 10% | 10 | 0.96 (0.84–1.11) | 0.58 | 82.4% | 0.13 | 0.0% |
| Study year, per 5 years | 10 | 1.43 (1.13–1.82) | 0.003 | 55.6% | 0.036 | 66.3% |
| Female, per 10% | 10 | 0.63 (0.32–1.22) | 0.17 | 80.8% | 0.12 | 0.0% |
| Baseline DAS28, per 1 unit | 10 | 1.32 (0.91–1.92) | 0.15 | 76.8% | 0.087 | 19.2% |

Outputs from random-effects pairwise meta-regression models evaluating study-level moderators of treatment effect heterogeneity for DAS28 remission attainment at 6 months, comparing: (i) b/tsDMARDs plus methotrexate vs. methotrexate alone; and (ii) b/tsDMARD monotherapy vs. methotrexate. Values greater than 1 indicate larger treatment effects with increasing values of the moderator. Residual I² and τ² represent heterogeneity remaining after inclusion of each moderator, while R² represents the proportion of between-study variance explained relative to the intercept-only model fitted to the same subset of studies (n). As such, residual heterogeneity estimates are not directly comparable across moderators with different numbers of contributing studies. RF: rheumatoid factor; CCP: anti-cyclic citrullinated peptide; MTX: methotrexate.

## **Supplementary Data.** Database search strategies.

***Ovid MEDLINE and Embase search strategy (database inception to 23 June 2026)***

**Search	Query**

1	rheumatoid arthritis.mp.

2	(etanercept or infliximab or adalimumab or certolizumab or golimumab or abatacept or rituximab or tocilizumab or sarilumab or tofacitinib or baricitinib or upadacitinib or filgotinib).mp.

3	1 and 2

4	randomized controlled trial.pt.

5	controlled clinical trial.pt.

6	clinical trial.pt.

7	4 or 5 or 6

8	3 and 7

***Cochrane Central Register of Controlled Trials (CENTRAL) search strategy (database inception to 23 June 2026)***

**Search	Query**

1	MeSH descriptor: [Arthritis, Rheumatoid] explode all trees

2	(etanercept or infliximab or adalimumab or certolizumab or golimumab or abatacept or rituximab or tocilizumab or sarilumab or tofacitinib or baricitinib or upadacitinib or filgotinib):ti,ab

3	1 and 2

## **References**

1.	Westhovens R, Robles M, Ximenes AC, et al. Clinical efficacy and safety of abatacept in methotrexate-naive patients with early rheumatoid arthritis and poor prognostic factors. Annals of the Rheumatic Diseases 2009;68(12):1870–1877. DOI: 10.1136/ard.2008.101121.

2.	St. Clair EW, Van Der Heijde DMFM, Smolen JS, et al. Combination of infliximab and methotrexate therapy for early rheumatoid arthritis: A randomized, controlled trial. Arthritis & Rheumatism 2004;50(11):3432–3443. DOI: 10.1002/art.20568.

3.	Emery P, Burmester GR, Bykerk VP, et al. Evaluating drug-free remission with abatacept in early rheumatoid arthritis: results from the phase 3b, multicentre, randomised, active-controlled AVERT study of 24 months, with a 12-month, double-blind treatment period. Annals of the Rheumatic Diseases 2015;74(1):19–26. DOI: 10.1136/annrheumdis-2014-206106.

4.	Emery P, Tanaka Y, Bykerk VP, et al. Sustained Remission and Outcomes with Abatacept plus Methotrexate Following Stepwise Dose De-escalation in Patients with Early Rheumatoid Arthritis. Rheumatology and Therapy 2023;10(3):707–727. DOI: 10.1007/s40744-022-00519-9.

5.	Emery P, Tanaka Y, Bykerk VP, et al. The trajectory of clinical responses in patients with early rheumatoid arthritis who achieve sustained remission in response to abatacept: subanalysis of AVERT-2, a randomized phase IIIb study. Arthritis Research & Therapy 2023;25(1). DOI: 10.1186/s13075-023-03038-2.

6.	Emery P, Bingham CO, Burmester GR, et al. Certolizumab pegol in combination with dose-optimised methotrexate in DMARD-naïve patients with early, active rheumatoid arthritis with poor prognostic factors: 1-year results from C-EARLY, a randomised, double-blind, placebo-controlled phase III study. Annals of the Rheumatic Diseases 2017;76(1):96–104. DOI: 10.1136/annrheumdis-2015-209057.

7.	Atsumi T, Yamamoto K, Takeuchi T, et al. The first double-blind, randomised, parallel-group certolizumab pegol study in methotrexate-naive early rheumatoid arthritis patients with poor prognostic factors, C-OPERA, shows inhibition of radiographic progression. Annals of the Rheumatic Diseases 2016;75(1):75–83. DOI: 10.1136/annrheumdis-2015-207511.

8.	Durez P, Malghem J, Toukap AN, et al. Treatment of early rheumatoid arthritis: A randomized magnetic resonance imaging study comparing the effects of methotrexate alone, methotrexate in combination with infliximab, and methotrexate in combination with intravenous pulse methylprednisolone. Arthritis & Rheumatism 2007;56(12):3919–3927. DOI: 10.1002/art.23055.

9.	Emery P, Breedveld FC, Hall S, et al. Comparison of methotrexate monotherapy with a combination of methotrexate and etanercept in active, early, moderate to severe rheumatoid arthritis (COMET): a randomised, double-blind, parallel treatment trial. The Lancet 2008;372(9636):375–382. DOI: 10.1016/s0140-6736(08)61000-4.

10.	Conaghan PG, Østergaard M, Bowes MA, et al. Comparing the effects of tofacitinib, methotrexate and the combination, on bone marrow oedema, synovitis and bone erosion in methotrexate-naive, early active rheumatoid arthritis: results of an exploratory randomised MRI study incorporating semiquantitative and quantitative techniques. Annals of the Rheumatic Diseases 2016;75(6):1024–1033. DOI: 10.1136/annrheumdis-2015-208267.

11.	Ducreux J, Durez P, Galant C, et al. Global Molecular Effects of Tocilizumab Therapy in Rheumatoid Arthritis Synovium. Arthritis & Rheumatology 2014;66(1):15–23. DOI: 10.1002/art.38202.

12.	Nam JL, Villeneuve E, Hensor EMA, et al. A randomised controlled trial of etanercept and methotrexate to induce remission in early inflammatory arthritis: the EMPIRE trial. Annals of the Rheumatic Diseases 2014;73(6):1027–1036. DOI: 10.1136/annrheumdis-2013-204882.

13.	Westhovens R, Rigby WFC, Van Der Heijde D, et al. Filgotinib in combination with methotrexate or as monotherapy versus methotrexate monotherapy in patients with active rheumatoid arthritis and limited or no prior exposure to methotrexate: the phase 3, randomised controlled FINCH 3 trial. Annals of the Rheumatic Diseases 2021;80(6):727–738. DOI: 10.1136/annrheumdis-2020-219213.

14.	Burmester GR, Rigby WF, Van Vollenhoven RF, et al. Tocilizumab in early progressive rheumatoid arthritis: FUNCTION, a randomised controlled trial. Annals of the Rheumatic Diseases 2016;75(6):1081–1091. DOI: 10.1136/annrheumdis-2015-207628.

15.	Emery P, Fleischmann RM, Moreland LW, et al. Golimumab, a human anti–tumor necrosis factor α monoclonal antibody, injected subcutaneously every four weeks in methotrexate‐naive patients with active rheumatoid arthritis: Twenty‐four–week results of a phase III, multicenter, randomized, double‐blind. Arthritis & Rheumatism 2009;60(8):2272–2283. DOI: 10.1002/art.24638.

16.	Emery P, Fleischmann RM, Hsia EC, Xu S, Zhou Y, Baker D. Efficacy of golimumab plus methotrexate in methotrexate-naïve patients with severe active rheumatoid arthritis. Clinical Rheumatology 2014;33(9):1239–1246. DOI: 10.1007/s10067-014-2731-y.

17.	Soubrier M, Puechal X, Sibilia J, et al. Evaluation of two strategies (initial methotrexate monotherapy vs its combination with adalimumab) in management of early active rheumatoid arthritis: data from the GUEPARD trial. Rheumatology 2009;48(11):1429–1434. DOI: 10.1093/rheumatology/kep261.

18.	Detert J, Bastian H, Listing J, et al. Induction therapy with adalimumab plus methotrexate for 24 weeks followed by methotrexate monotherapy up to week 48 versus methotrexate therapy alone for DMARD-naïve patients with early rheumatoid arthritis: HIT HARD, an investigator-initiated study. Annals of the Rheumatic Diseases 2013;72(6):844–850. DOI: 10.1136/annrheumdis-2012-201612.

19.	Takeuchi T, Yamanaka H, Ishiguro N, et al. Adalimumab, a human anti-TNF monoclonal antibody, outcome study for the prevention of joint damage in Japanese patients with early rheumatoid arthritis: the HOPEFUL 1 study. Annals of the Rheumatic Diseases 2014;73(3):536–543. DOI: 10.1136/annrheumdis-2012-202433.

20.	Yamanaka H, Ishiguro N, Takeuchi T, et al. Recovery of clinical but not radiographic outcomes by the delayed addition of adalimumab to methotrexate-treated Japanese patients with early rheumatoid arthritis: 52-week results of the HOPEFUL-1 trial. Rheumatology 2014;53(5):904–913. DOI: 10.1093/rheumatology/ket465.

21.	Nam JL, Villeneuve E, Hensor EMA, et al. Remission induction comparing infliximab and high-dose intravenous steroid, followed by treat-to-target: a double-blind, randomised, controlled trial in new-onset, treatment-naive, rheumatoid arthritis (the IDEA study). Annals of the Rheumatic Diseases 2014;73(1):75–85. DOI: 10.1136/annrheumdis-2013-203440.

22.	Tak PP, Rigby WF, Rubbert-Roth A, et al. Inhibition of joint damage and improved clinical outcomes with rituximab plus methotrexate in early active rheumatoid arthritis: the IMAGE trial. Annals of the Rheumatic Diseases 2011;70(1):39–46. DOI: 10.1136/ard.2010.137703.

23.	Bejarano V, Quinn M, Conaghan PG, et al. Effect of the early use of the anti–tumor necrosis factor adalimumab on the prevention of job loss in patients with early rheumatoid arthritis. Arthritis Care & Research 2008;59(10):1467–1474. DOI: 10.1002/art.24106.

24.	Østergaard M, Van Vollenhoven RF, Rudin A, et al. Certolizumab pegol, abatacept, tocilizumab or active conventional treatment in early rheumatoid arthritis: 48-week clinical and radiographic results of the investigator-initiated randomised controlled NORD-STAR trial. Annals of the Rheumatic Diseases 2023;82(10):1–10. DOI: 10.1136/ard-2023-224116.

25.	Hetland ML, Haavardsholm EA, Rudin A, et al. Active conventional treatment and three different biological treatments in early rheumatoid arthritis: phase IV investigator initiated, randomised, observer blinded clinical trial. bmj 2020;371.

26.	Hørslev-Petersen K, Hetland ML, Junker P, et al. Adalimumab added to a treat-to-target strategy with methotrexate and intra-articular triamcinolone in early rheumatoid arthritis increased remission rates, function and quality of life. The OPERA Study: an investigator-initiated, randomised, double-blind. Annals of the Rheumatic Diseases 2014;73(4):654–661. DOI: 10.1136/annrheumdis-2012-202735.

27.	Kavanaugh A, Fleischmann RM, Emery P, et al. Clinical, functional and radiographic consequences of achieving stable low disease activity and remission with adalimumab plus methotrexate or methotrexate alone in early rheumatoid arthritis: 26-week results from the randomised, controlled OPTIMA study. Annals of the Rheumatic Diseases 2013;72(1):64–71. DOI: 10.1136/annrheumdis-2011-201247.

28.	Lee EB, Fleischmann R, Hall S, et al. Tofacitinib versus Methotrexate in Rheumatoid Arthritis. New England Journal of Medicine 2014;370(25):2377–2386. DOI: 10.1056/NEJMoa1310476.

29.	Breedveld FC, Weisman MH, Kavanaugh AF, et al. The PREMIER study: A multicenter, randomized, double‐blind clinical trial of combination therapy with adalimumab plus methotrexate versus methotrexate alone or adalimumab alone in patients with early, aggressive rheumatoid arthritis who had not had previous methotrexate treatment. Arthritis & Rheumatism 2006;54(1):26–37. DOI: 10.1002/art.21519.

30.	Keystone EC, Haraoui B, Guérette B, Mozaffarian N, Liu S, Kavanaugh A. Clinical, Functional, and Radiographic Implications of Time to Treatment Response in Patients With Early Rheumatoid Arthritis: a Posthoc Analysis of the PREMIER Study. The Journal of Rheumatology 2014;41(2):235–243. DOI: 10.3899/jrheum.121468.

31.	Fleischmann R, Schiff M, Van Der Heijde D, et al. Baricitinib, Methotrexate, or Combination in Patients With Rheumatoid Arthritis and No or Limited Prior Disease‐Modifying Antirheumatic Drug Treatment. Arthritis & Rheumatology 2017;69(3):506–517. DOI: 10.1002/art.39953.

32.	Van Vollenhoven R, Takeuchi T, Pangan AL, et al. Efficacy and Safety of Upadacitinib Monotherapy in Methotrexate‐Naive Patients With Moderately‐to‐Severely Active Rheumatoid Arthritis (SELECT‐EARLY): A Multicenter, Multi‐Country, Randomized, Double‐Blind, Active Comparator–Controlled Trial. Arthritis & Rheumatology 2020;72(10):1607–1620. DOI: 10.1002/art.41384.

33.	Van Vollenhoven R, Strand V, Takeuchi T, et al. Upadacitinib monotherapy versus methotrexate monotherapy in patients with rheumatoid arthritis: efficacy and safety through 5 years in the SELECT-EARLY randomized controlled trial. Arthritis Research & Therapy 2024;26(1). DOI: 10.1186/s13075-024-03358-x.

34.	Bijlsma JWJ, Welsing PMJ, Woodworth TG, et al. Early rheumatoid arthritis treated with tocilizumab, methotrexate, or their combination (U-Act-Early): a multicentre, randomised, double-blind, double-dummy, strategy trial. The Lancet 2016;388(10042):343–355. DOI: 10.1016/S0140-6736(16)30363-4.

35.	Emery P, Horton S, Dumitru RB, et al. Pragmatic randomised controlled trial of very early etanercept and MTX versus MTX with delayed etanercept in RA: the VEDERA trial. Annals of the Rheumatic Diseases 2020;79(4):464–471. DOI: 10.1136/annrheumdis-2019-216539.
